# Binasal occlusion for the non-invasive management of acute acquired comitant esotropia

**DOI:** 10.3389/fmed.2026.1742695

**Published:** 2026-03-09

**Authors:** Ruiying Li, Ruoshi Li, Xiaoqing Li

**Affiliations:** 1 Department of Ophthalmology, Peking University First Hospital, Beijing, China; 2 Peking University Pediatric Vision Research Center, Beijing, China

**Keywords:** acute acquired comitant esotropia, binasal occlusion, binocular vision, conservative therapy, diplopia

## Abstract

**Purpose:**

To assess the clinical effectiveness of binasal occlusion (BNO) as a non-invasive treatment for acute acquired comitant esotropia (AACE) and to explore potential predictors of therapeutic response.

**Methods:**

This retrospective case series included 32 AACE patients who underwent BNO therapy for at least 6 months. Changes in ocular deviation angles, diplopia resolution, and binocular function were assessed before and after treatment. Patients were categorized into cured (orthophoria), effective [≥10 prism diopter (PD) reduction], and ineffective groups. Univariate and multivariate analyses were conducted to identify predictors of therapeutic success.

**Results:**

BNO significantly reduced ocular deviation. The median near deviation decreased from 25.00 PD (IQR 20.00–30.00) to 17.50 PD (IQR 5.50–33.75) (*P* = 0.002), and the median distance deviation decreased from 30.00 PD (IQR 21.25–40.00) to 20.00 PD (IQR 5.25–35.00) (*P* < 0.001). Diplopia resolved in 50% of patients (*P* < 0.001), and fusion function improved significantly (*P* = 0.019). Smaller initial deviations and shorter disease duration were associated with favorable outcomes in univariate analysis but did not emerge as independent predictors in multivariate analysis, suggesting a potential synergistic interaction.

**Conclusion:**

BNO represents a promising, non-invasive intervention for AACE, particularly in cases with smaller deviations and early presentation. This case series provides preliminary evidence that BNO may reduce deviation and potentially lower surgical need. However, the absence of a control group precludes causal inference. Future randomized controlled trials with standardized outcomes are needed to confirm efficacy and define BNO's role in AACE management.

## 1 Introduction

Acute acquired comitant esotropia (AACE) is a rare but increasingly recognized form of strabismus characterized by a sudden onset of esodeviation and diplopia in individuals with previously normal binocular vision (1). Unlike congenital or accommodative esotropia, AACE presents without significant refractive errors or neurological symptoms in most cases, though a subset of patients may have underlying central nervous system abnormalities such as intracranial tumors, hydrocephalus, or demyelinating diseases (2). The exact etiology remains unclear, but proposed mechanisms include excessive near work, prolonged screen exposure, decompensated esophoria, and potential neurogenic factors (3, 4). There has been a notable increase in incidence among children during the COVID-19 pandemic (5). AACE is commonly classified as Type I (Swan, normal refraction), Type II (Burian-Franceschetti, uncorrected myopia), or Type III (Bielschowsky, visual deprivation) (6).

Treatment of AACE remains challenging due to variability in presentation and response to therapy. Optical correction, prisms, and occlusion therapy have been used to alleviate symptoms, but their effectiveness varies depending on the degree of esodeviation and fusional reserves. Neuroimaging should be considered in AACE with atypical features or poor treatment response to rule out neurologic causes (7). Botulinum toxin injections and strabismus surgery remain the primary options for persistent or severe cases, but both involve potential risks and recurrence (8–10). Given these limitations, there is growing interest in non-invasive therapies that may promote ocular realignment and improve binocular function without surgical intervention.

Binasal occlusion (BNO) has been proposed as a non-invasive treatment for AACE, with potential benefits in improving ocular alignment and fusion. By occluding the nasal visual fields, BNO is believed to reduce visual confusion, alleviate diplopia, and encourage fusional divergence by reducing the conflicting nasal retinal disparity cues that drive excessive convergence, thereby allowing the fusional vergence system to re-establish a more divergent posture (11). Unlike full occlusion, BNO maintains partial peripheral vision, which may help sustain binocular interaction and prevent deep suppression. Previous studies suggest that BNO can facilitate alternating fixation, reduce anomalous retinal correspondence, and support long-term stabilization of ocular alignment (12). However, despite these theoretical advantages, clinical evidence regarding BNO's efficacy in AACE remains limited, and its precise role in treatment is not well defined.

Therefore, this study aimed to assess the clinical effectiveness of BNO in the management of AACE, focusing on its impact on ocular alignment, diplopia resolution, and binocular function. We also explored potential predictors of treatment success to better inform patient selection and therapeutic decision-making.

## 2 Methods

### 2.1 Study design and eligibility

This retrospective case series was conducted at the Department of Pediatric Ophthalmology, Peking University First Hospital, and included patients who underwent BNO therapy between September 2020 and December 2023. All participants had a minimum follow-up of 6 months. This retrospective study was approved by the Ethics Committee of Peking University First Hospital (No. 2022SF46), which waived the requirement for informed consent. All clinical images have been professionally de-identified to ensure patient anonymity.

Patients with acute-onset comitant esotropia, a deviation difference of ≤ 5 prism diopters (PD) across all gaze directions, full extraocular motility, and persistent or intermittent diplopia were included. Eligibility required no underlying systemic diseases, confirmed through neurological and endocrinological evaluations, including cranial MRI and endocrine assessments, and a best-corrected visual acuity (BCVA) of ≥20/25 in both eyes. Patients were excluded if they had a history of strabismus, amblyopia, ophthalmic surgery, neurological or systemic disorders (e.g., intracranial lesions, thyroid dysfunction, or diabetes), or incomplete medical records or loss to follow-up.

These selected AACE patients all underwent a comprehensive ophthalmic examination before BNO treatment. Cycloplegic refraction was performed using 1% atropine sulfate for children under 7 years and 0.5% compound tropicamide for those 7 years and older. As the accommodative convergence/accommodation (AC/A) ratio was not part of our standard diagnostic protocol for AACE, it was not routinely assessed; however, fusional vergence was evaluated in all patients. Myopic patients received the lowest power correction for best-corrected visual acuity, hyperopic patients were fully corrected, and emmetropic patients were prescribed plano lenses for BNO application. Deviation was measured using the prism alternating cover test (PACT); diplopia was assessed with the Worth 4-dot test; motor fusion was evaluated with the synoptophore; and stereopsis was tested with the Titmus stereotest, considering stereopsis below 100 arc seconds as normal. Treatment outcomes were classified using predefined criteria. Orthophoria was defined as a residual deviation of ≤ 5 PD at near and distance without BNO, a threshold commonly regarded as clinically acceptable alignment. An effective outcome was defined as a deviation reduction of ≥10 PD, representing a meaningful and clinically perceptible improvement beyond measurement variability, whereas reductions of < 10 PD were classified as ineffective. Additionally, we documented whether patients subsequently required prism correction or proceeded to surgery during the follow-up period.

### 2.2 Binasal occlusion (BNO)

**Figure 1:**
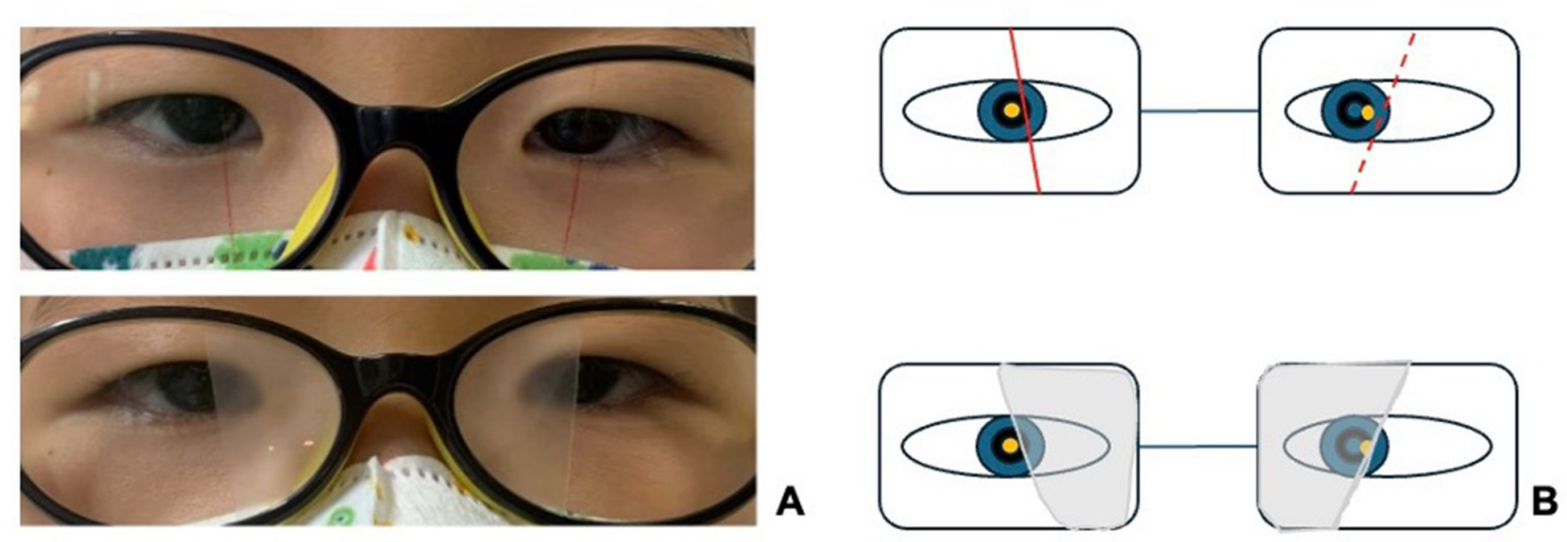
BNO application **(A)** Clinical photograph. Relative to the CLR, the occlusion border is positioned nasally for the fixating right eye and temporally for the deviating left eye. **(B)** Schematic illustration. The CLR (yellow dot) serves as the reference point. The depressive membrane is illustrated as an upper-wide and lower-narrow shape. A solid red line indicates the nasalward position of the fixating eye's border, and a dashed red line indicates the temporalward position of the deviating eye's border.

BNO was applied after appropriate refractive correction, with the fixating and deviating eyes identified using a point light source at 33 cm. A nasal occlusion strip, designed with an upper-wide and lower-narrow shape tilted 10–15° downward, was positioned so that the fixating eye's occlusion edge aligned nasally with the corneal light reflex (CLR), while the deviating eye's occlusion edge aligned temporally with the CLR (as depicted in Figures 1A, B). Customized occlusion films were cut and affixed to the lenses accordingly, and post-application alignment was reassessed to ensure proper positioning. Patients were instructed to wear BNO spectacles during waking hours, primarily for near work. Monthly follow-ups assessed compliance and adjusted occlusion width based on alignment changes. Treatment was tapered if orthophoria ( ≤ 5 PD) was maintained for three consecutive months without diplopia.

### 2.3 Statistical analyses

Data were analyzed using SPSS 26.0 with appropriate statistical descriptors: normally distributed continuous variables were expressed as mean ± standard deviation (SD), non-normally distributed variables as the median with interquartile range [M (IQR)], and categorical variables as percentages or constituent ratios. Treatment effects were assessed through Wilcoxon signed-rank tests for paired non-parametric comparisons and McNemar's tests for categorical changes. Intergroup outcome differences were evaluated using Kruskal–Wallis *H*-tests, one-way ANOVA, or Fisher's exact tests, depending on data characteristics. Given the sample size and three outcome categories, the study was likely underpowered for multivariate modeling; thus, these analyses should be considered exploratory. Variables showing significance in univariate analyses (*P* < 0.05) were incorporated into multivariate logistic regression models to identify independent predictors of therapeutic efficacy. A *P*-value < 0.05 was used to define statistical significance throughout the analysis.

## 3 Results

### 3.1 Participant characteristics

The study cohort consisted of 32 AACE patients (21 males, 65.6%; 11 females) treated with BNO. Participants had a mean age of 15.8 ± 11.8 years (range 4–42) and a mean disease duration of 23.0 ± 40.0 months (range 0.5–180). Given the wide age range, we performed a subgroup analysis comparing children ( ≤ 18 years, *n* = 23) and adults (>18 years, *n* = 9). No significant differences in baseline deviation or treatment response were found between these groups (all *P* > 0.05). Refractive analysis identified 22 myopic cases (68.8%), including 15 with moderate-high myopia [spherical equivalent (SE) ≤ – 3.00 D] and 7 with mild myopia (−3.00 D < SE ≤ – 0.50 D), alongside 4 emmetropic patients (12.5%) and 6 with low-moderate hyperopia (SE ≥ + 0.75 D, 18.8%). Ocular deviation patterns varied significantly: 16 patients (50.0%) exhibited comparable near-distance deviations ( ≤ 5 PD difference), 4 (12.5%) demonstrated near-dominant deviations (≥10 PD increase at near), and 12 (37.5%) showed distance-dominant deviations (≥10 PD increase at distance). Etiological assessment revealed prolonged near-work (>4 h/day) in 30 patients (93.7%), while two cases (6.3%) were associated with documented psychological stressors. Of the 32 patients, 30 reported persistent diplopia at baseline; the remaining two had intermittent diplopia that was not present at the initial examination.

### 3.2 Changes in ocular deviation after BNO therapy

BNO significantly reduced both near and distance deviations in patients with AACE (Table 1). The median near deviation decreased from 25.00 PD (IQR 20.00–30.00) to 17.50 PD (IQR 5.50–33.75) (*Z* = −3.058, *P* = 0.002), while the median distance deviation declined from 30.00 PD (IQR 21.25–40.00) to 20.00 PD (IQR 5.25–35.00) (*Z* = −3.567, *P* < 0.001). These reductions highlight BNO's efficacy in improving motor alignment. Clinically, this improvement translated to nine patients (28.1%) avoiding further surgical or prism intervention during follow-up.

**Table 1:** Changes in ocular deviation after BNO therapy [M (IQR), PD].

**Measurement distance**	**Before BNO**	**After BNO**	***Z*-value**	***P***
PACT at near	25.00 (20.00–30.00)	17.50 (5.50–33.75)	−3.058	0.002
PACT at distance	30.00 (21.25–40.00)	20.00 (5.25–35.00)	−3.567	< 0.001

Wilcoxon signed-rank test; PD, prism diopters; M (IQR), median (interquartile range).

### 3.3 Changes in binocular function after BNO therapy

BNO therapy induced significant improvements in binocular function (Table 2). Motor fusion rates increased from 50.0% (16/32) to 78.1% (25/32) (*P* = 0.019). Of the 32 patients, 30 presented with persistent diplopia at baseline; in this subgroup, diplopia resolved in 50.0% (15/30, *P* < 0.001). However, Titmus stereopsis showed no statistically significant improvement (31.3%−46.9%, *P* = 0.200), suggesting that BNO primarily enhances fusion rather than stereoscopic function.

**Table 2:** Changes in binocular function after BNO therapy *n* (%).

**Binocular function**	**Before BNO**	**After BNO**	**χ^2^**	***P***
Motor fusion			5.497	0.019
Present	16 (50.0)	25 (78.1)		
Absent	16 (50.0)	7 (21.9)		
Titmus stereopsis			1.641	0.200
Present	10 (31.3)	15 (46.9)		
Absent	22 (68.8)	17 (53.1)		
Diplopia			20.000	< 0.001
Present	30 (100.0)	15 (50.0)		
Absent	0 (0.0)	15 (50.0)		

McNemar's test.

### 3.4 Predictive factors for treatment response

In the univariate analysis (Table 3), baseline near deviation (*F* = 13.249, *P* = 0.001), distance deviation (*F* = 11.359, *P* = 0.003), and shorter disease duration (*H* = 8.399, *P* = 0.015) were significantly associated with therapeutic success. Patients who achieved complete remission exhibited smaller baseline deviations and a shorter duration of disease. Subgroup analysis of patients with disease duration ≤ 3 months (*n* = 12) showed a significantly higher cure rate compared to those with longer duration (50.0 vs. 10.0%, *P* = 0.03). In the multivariate analysis (Table 4), none of these factors independently predicted treatment success (near deviation *OR* = 0.85, 95% CI 0.66–1.10; distance deviation *OR* = 0.89, 95% CI 0.69–1.15; disease duration *OR* = 0.84, 95% CI 0.68–1.04; all *P* > 0.05). This discrepancy suggests that the observed association may reflect a synergistic interaction between smaller baseline deviation and early intervention, rather than independent predictive value. The limited sample size likely contributed to the lack of significance in the multivariate model.

**Table 3:** Univariate analysis of factors associated with BNO efficacy.

**Parameters**	**Ineffective (*n* = 15)**	**Effective (*n* = 9)**	**Cured (*n* = 8)**	***H*/χ^2^/*F***	***P***
PACT at near (PD)	25.00 ± 10.35	47.78 ± 22.65	17.50 ± 8.02	13.249	0.001^a^
PACT at distance (PD)	30.67 ± 11.93	48.33 ± 22.08	19.63 ± 9.98	11.359	0.003^a^
Age (years)	12.00 (10.00, 24.00)	9.00 (5.50, 24.50)	11.50 (7.25, 34.25)	0.673	0.714^a^
Disease duration (months)	12.00 (6.00, 60.00)	7.00 (2.50, 12.00)	2.50 (0.625, 3.75)	8.399	0.015^a^
**Refractive error (SE, D)**			
OD	−2.21 ± 2.71	−1.72 ± 3.27	−2.32 ± 2.92	0.109	0.897^b^
OS	−2.14 ± 2.81	−0.99 ± 3.17	−2.12 ± 3.06	0.483	0.622^b^
Sex *n* (%)				0.973	0.639^c^
Male	11 (73.3)	5 (55.6)	5 (62.5)		
Female	4 (26.7)	4 (44.4)	3 (37.5)		
Titmus stereopsis				4.963	0.092^c^
Yes	4 (26.7)	1 (11.1)	5 (62.5)		
No	11 (73.3)	8 (88.9)	3 (37.5)		
Motor fusion				1.247	0.660^c^
Yes	9 (60.0)	4 (44.4)	3 (37.5)		
No	6 (40.0)	5 (55.6)	5 (62.5)		
Ocular deviation patterns				5.919	0.180^c^
Near-distance	6 (40.0)	7 (77.8)	3 (37.5)		
Near-dominant	1 (6.7)	1 (11.1)	2 (25.0)		
Distance-dominant	8 (53.3)	1 (11.1)	3 (37.5)		

SE, mean spherical equivalents; PD, prism diopters; OD/OS, right/left eye; D, diopter; SD, standard deviation.

^a^Kruskal–Wallis test.

^b^One-way ANOVA.

^c^Fisher's exact test.

**Table 4:** Multivariate logistic regression for BNO efficacy.

**Parameters**	**Effective vs. ineffective**	***P***	**Cured vs. ineffective**	***P***
	**OR (95% CI)**		**OR (95% CI)**	
PACT at near (PD)	1.11 (0.98–1.27)	0.096	0.85 (0.66–1.10)	0.214
PACT at distance (PD)	0.99 (0.88–1.12)	0.927	0.89 (0.69–1.15)	0.364
Disease duration (months)	0.98 (0.93–1.03)	0.383	0.84 (0.68–1.04)	0.111

Multinomial logistic regression; Reference group: Ineffective.

## 4 Discussion

This study evaluated the efficacy of BNO therapy in patients with AACE and explored potential predictors of treatment response. The results showed that BNO significantly reduced ocular deviation and improved motor fusion, particularly in patients with lower baseline deviation and shorter disease duration ( ≤ 3 months). These findings support the role of BNO as an effective conservative intervention for esotropia, consistent with previous reports (11). However, as an uncontrolled retrospective case series, this study cannot definitively attribute the observed improvements to BNO alone, given the potential for spontaneous improvement or regression to the mean in early AACE. While promising, our findings require validation in controlled trials. Although univariate analysis suggested that baseline deviation and disease duration may influence treatment outcomes, multivariate analysis did not identify any independent predictors, possibly due to confounding interactions or insufficient sample size to detect subtle effects. Thus, our findings are hypothesis-generating, suggesting a potential synergy between early intervention and milder deviation rather than definitive predictive rules.

Our outcome categories used conventional prism diopter thresholds. A ≥10 PD reduction (“effective”) represents a clinically meaningful change, associated with functional gains. Importantly, nine patients (28.1%) avoided further intervention, highlighting that partial deviation reduction with functional improvement can alter clinical management.

The precise mechanism by which BNO exerts its therapeutic effects is still under investigation. Theoretical models suggest that nasal field occlusion alleviates sensory confusion and interocular suppression, thereby enhancing fusional divergence and stabilizing ocular alignment (12–16). In our study, patients demonstrated significant improvement in fusion function following BNO, thus supporting this hypothesis. However, stereopsis did not show significant improvement. This may be attributable to individual variations in fusional reserves and the presence of pre-existing sensory adaptations, such as anomalous retinal correspondence (ARC), which can develop with prolonged misalignment and potentially limit the recovery of stereopsis (17, 18).

Univariate analysis demonstrated that lower baseline esodeviation and shorter disease duration were significantly associated with superior treatment outcomes. Patients presenting with smaller initial deviations were more likely to achieve resolution, indicating that BNO may be more effective in cases where fusional mechanisms remain intact. Similarly, a shorter disease duration was correlated with enhanced outcomes, underscoring the critical role of early intervention in mitigating maladaptive sensory changes (19, 20). However, multivariate analysis did not confirm these factors as independent predictors, suggesting that other unmeasured variables—such as vergence adaptability or neural plasticity—may play a significant role in BNO efficacy. Comparable challenges in identifying robust independent predictors have been documented in studies on prism adaptation and occlusion therapy, further emphasizing the intricate nature of binocular vision adaptation (21, 22). Future investigations employing larger sample sizes and objective assessments of fusional reserves may elucidate these relationships more clearly.

Currently, treatment options for AACE include prisms, botulinum toxin injections, and surgery. Prisms are useful in mild cases but are ineffective for large-angle esotropia (23, 24). Botulinum toxin provides a minimally invasive option, but its recurrence rate is high (8). Surgery remains the definitive treatment for persistent AACE, but outcomes can be unpredictable, particularly in cases with fluctuating deviations or evolving sensory adaptations (25, 26). Compared to these modalities, BNO is a non-invasive and easily applicable intervention that may help stabilize binocular function and reduce surgical need. While it may not be effective in all cases, particularly those with severe misalignment or longstanding adaptations, its potential as a first-line conservative therapy warrants further exploration. In our cohort, 28.1% of patients avoided further intervention, suggesting a possible role for BNO in delaying or preventing more invasive procedures.

This study has limitations, including its uncontrolled retrospective design, modest sample size, and heterogeneous cohort, which preclude definitive causal conclusions about BNO's efficacy relative to the natural history of AACE. However, it provides essential preliminary data on treatment effect and identifies a potential responder profile (smaller deviation, shorter duration), thereby informing the design of future prospective randomized controlled trials.

## 5 Conclusion

BNO therapy constitutes a promising conservative intervention for AACE in this case series, markedly reducing ocular deviation while enhancing fusional function. Smaller baseline deviations and shorter disease duration ( ≤ 3 months) synergistically predicted therapeutic success rather than independently. These findings highlight BNO as a viable first-line, non-invasive option to explore, particularly in milder, early presenting cases. However, the lack of a control group necessitates caution in interpreting efficacy. Future randomized controlled trials are warranted to confirm efficacy and define BNO's role in AACE management.

## Data Availability

The original contributions presented in the study are included in the article/supplementary material, further inquiries can be directed to the corresponding author.
